# The Efficiency of Systemic Dapsone in the Treatment of Acne Fulminans: A Clinical Case Report

**DOI:** 10.7759/cureus.83234

**Published:** 2025-04-30

**Authors:** Asmaa Lahrougui, Maryem Aboudourib, Layla Bendaoud, Said Amal, Ouafa Hocar

**Affiliations:** 1 Department of Dermatology, Mohammed VI University Hospital, Marrakech, MAR; 2 Department of Dermatology and Venerology, Mohammed VI University Hospital, Marrakech, MAR

**Keywords:** dapsone, isotretinoin, isotretinoin resistance, severe acne, systemic dapsone

## Abstract

Acne is a benign, complex, multifactorial disease resulting from hormonal, genetic, and environmental factors. It is considered a pubertal phenomenon and mainly affects adolescents, although some atypical cases can be observed in newborns and adults. Diagnosis is often straightforward, but in some cases, management can be difficult, with dramatic psychological and social consequences. Due to its antibiotic and immunomodulatory effects, dapsone has become an indispensable treatment in dermatology, particularly for severe acne resistant to conventional therapies. We report the case of a 31-year-old patient with acne fulminans, resistant to oral corticosteroids and isotretinoin, who was treated with 100 mg/day of dapsone, resulting in complete resolution of acne after six months of treatment. No side effects or recurrences were observed after three years of semi-annual follow-up.

## Introduction

Acne fulminans is a rare severe form of acne that affects mainly adolescent males with a history of moderate-to-severe acne vulgaris. Clinically, it manifests as intensely painful nodules and hemorrhagic plaques that progress to severe necrotic ulcers, often resulting in extensive scarring, and is associated with systemic symptoms such as fever and arthralgia. Diagnosis is based on clinical assessment, diagnostic tests including blood tests, cultures to exclude other infections, and skin biopsy in cases of diagnostic doubt. The differential diagnosis mainly includes acne conglobata, which is more chronic without associated systemic signs, and rosacea fulminans, which preferentially affects women without a history of acne or associated systemic signs. Early and appropriate multidisciplinary management is essential to prevent the serious complications of acne fulminans, such as bone and joint infections and severe scarring, which can affect the patient's mental health [1,2].

Treatment recommendations are mainly based on a combination of corticosteroids and isotretinoin, although cases of isotretinoin-induced acne fulminans and therapeutic resistance have been reported, highlighting the importance of regular follow-up [1]. In such cases, dapsone has been shown to be effective in severe acne due to its antibiotic and immunomodulatory effects. Its use has also been shown to be effective in various inflammatory and autoimmune skin conditions such as dermatitis herpetiformis, linear IgA bullous dermatosis, lupus erythematosus, and vasculitis, making it an interesting therapeutic option in dermatology [3].

We report a case of acne fulminans resistant to oral corticosteroids and isotretinoin that was completely cured after six months of treatment with dapsone, without any recurrence after three years of regular clinical and biological follow-up.

## Case presentation

**Figure 1:**
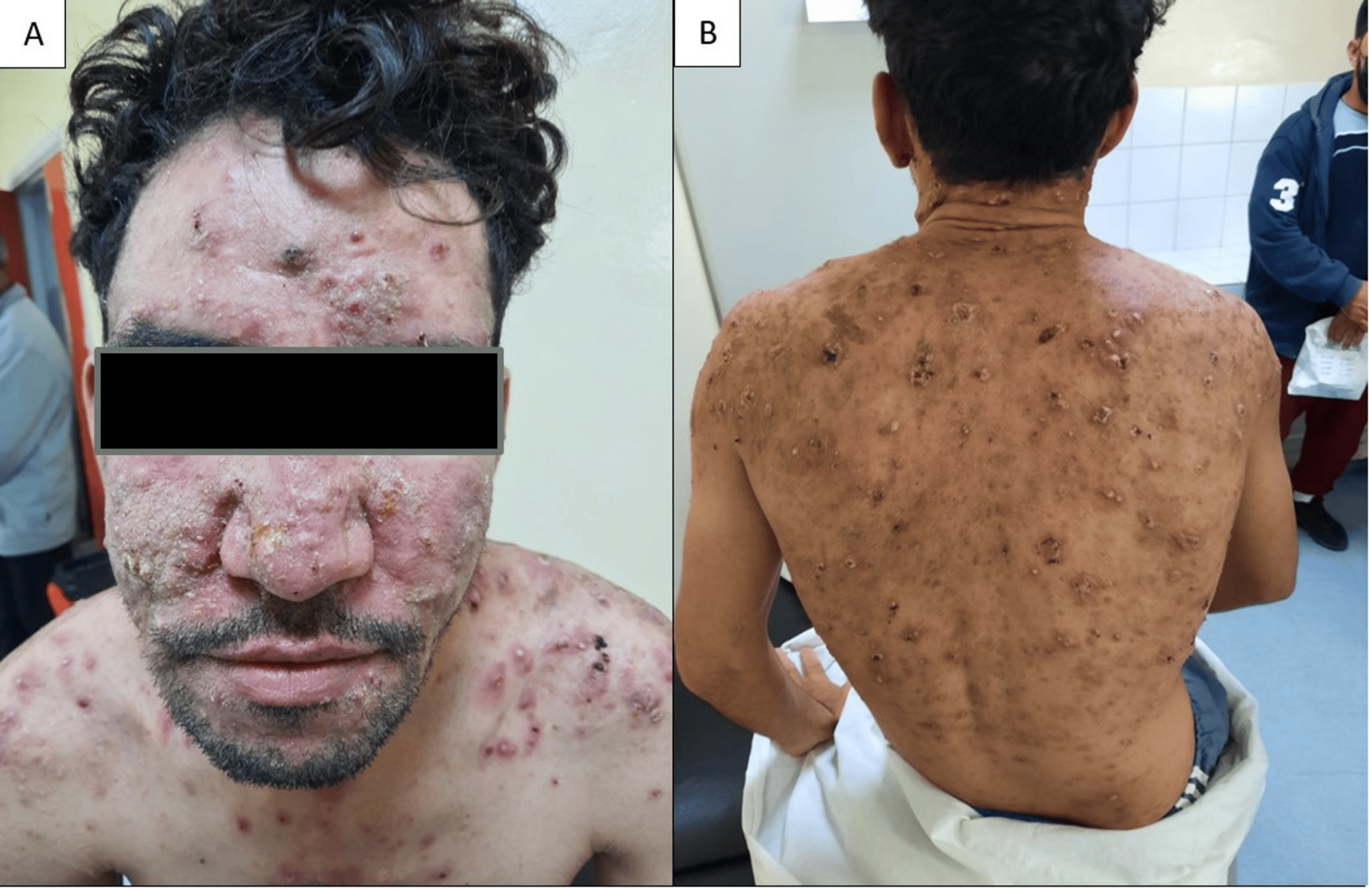
(a, b) Diffuse hyperkeratotic pustular-nodular lesions on the face, trunk, and back, associated with atrophic scarring, and ulcero-necrotic and hemorrhagic lesions.

A 31-year-old male patient with acne conglobata since the age of 14, treated with a total of three courses of isotretinoin (150mg/kg/course) with slight clinical improvement, was consulted because of a painful, non-pruritic eruption on the face, back, and trunk of one month's duration, associated with polyarthralgia of the knees and dorsolumbar spine, and a change in his general condition and quality of life. Dermatological examination revealed diffuse hyperkeratotic pustular-nodular lesions on the face, trunk, and back, associated with atrophic scarring, and ulcero-necrotic and hemorrhagic lesions (Figure 1).

The biological examination revealed leukocytosis with a white blood cell count of 16,000 elements/mm^3^ and a C-reactive protein level of 124 mg/L, a cytobacteriological examination of the urine (CBEU) did not reveal any microorganisms, bacteriological sampling of the pustules showed the presence of Corynebacterium amycolatum, and the glucose-6-phosphate dehydrogenase deficiency (G6PD) levels were normal (Table 1).

**Table 1:** Biological test results MCV, mean corpuscular volume; MCH, mean corpuscular hemoglobin; MCHC, mean corpuscular hemoglobin concentration; G6PD, glucose-6-phosphate dehydrogenase deficiency; C-reactive protein

	Patient value	Reference value
Leukocytes	16,760/uL	4.00–11.00x10^3^/uL
Neutrophils	6,500/uL	1.40–7.70x10^3^/uL
Eosinophilic polymorphs	230/uL	0.02–0.63x10^3^/uL
Basophilic polynuclear cells	3/uL	0.00–0.11x10^3^/uL
Lymphocytes	2,140/uL	1.00–4.8010^3^/uL
Monocytes	702/uL	0.18–1.00x10^3^/uL
Red blood cells	4,400/uL	4.28–6.00x10^6^/uL
Hemoglobin	13.2 g/dL	13.0–18.0 g/dL
Hematocrit	39.4%	39.0–53.0%
MCV	86.8 fL	78.0–98.0 fL
MCH	29.5 pg	26.0–34.0 pg
MCHC	34 g/dL	31.0–36.5 g/dL
Platelets	317,000/uL	150–450x10^3^/uL
G6PD	589 U/L	500–1,500 U/L
CRP	124 mg/L	0–5 mg/L

**Figure 2:**
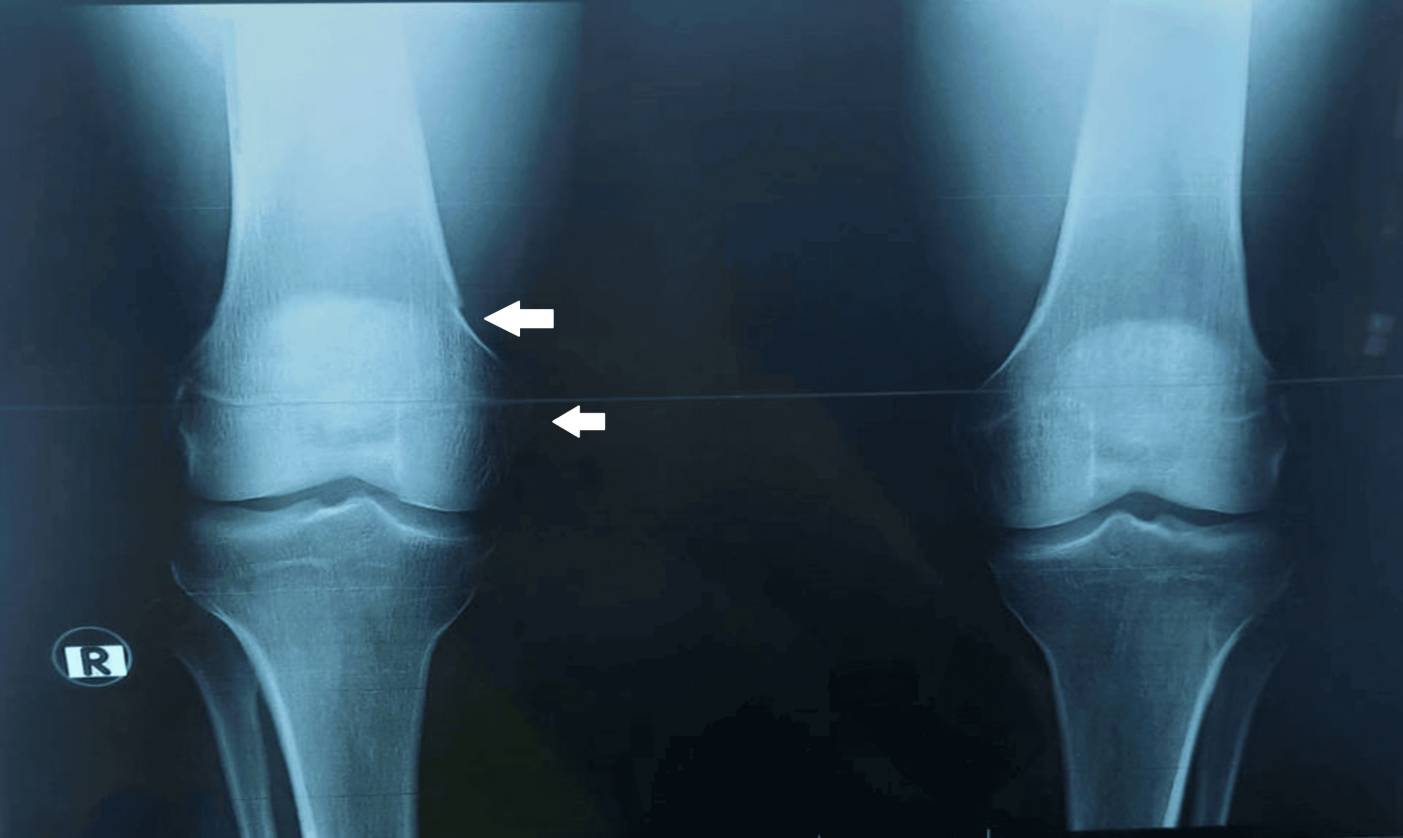
X-ray of the right knee showing osteolysis of the medial condyle.

Standard X-rays of the thorax, hands, and dorsolumbar spine were normal, while X-rays of the right knee revealed osteolysis of the medial condyle (Figure 2).

**Figure 3:**
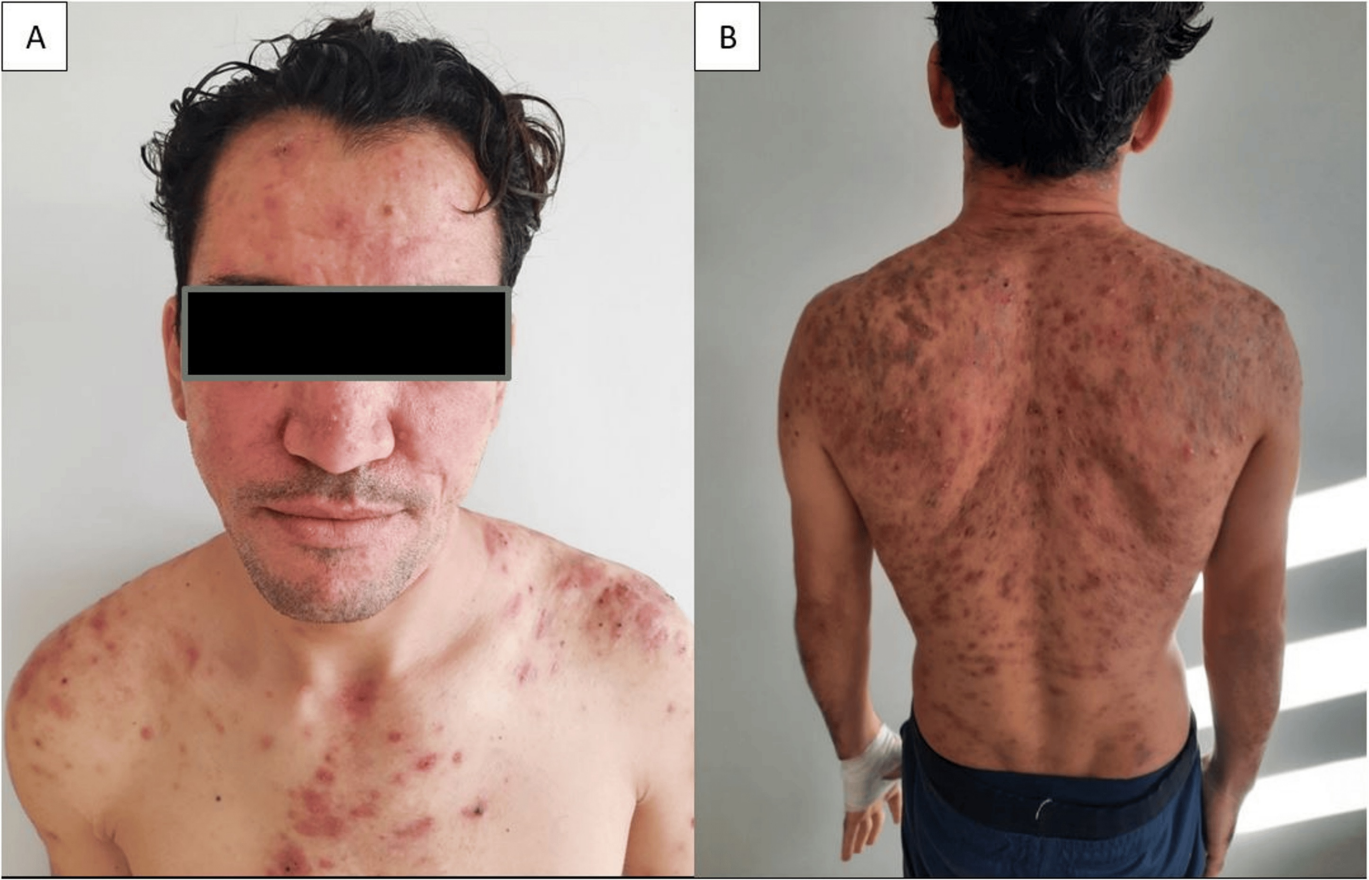
Clinical improvement after two months of dapsone treatment.

**Figure 4:**
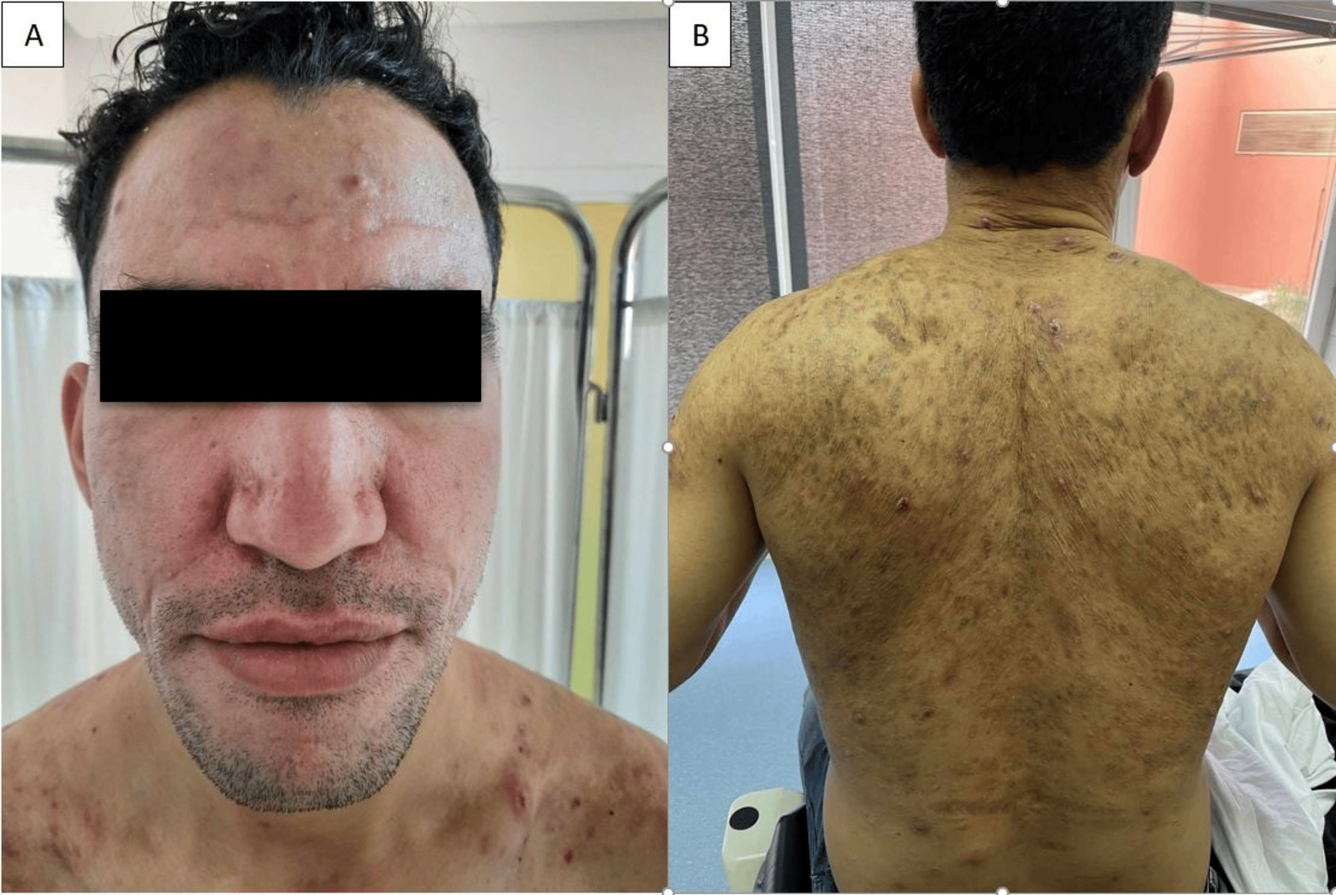
Total clinical improvement after six months of dapsone treatment.

Given this clinical, biological, and radiological aspect, the diagnosis of acne fulminans was made, and our patient was initially treated with the combination of oral prednisone (1mg/kg/day) and low-dose of isotretinoin (0.1mg/kg/day); however, given the lack of therapeutic response, we replaced the current treatments with dapsone 100mg daily for six months. Rigorous biological monitoring showed normal levels of methemoglobinemia, complete blood count with reticulocyte count, and liver and renal function tests. The clinical course showed a significant improvement starting from the first week, with partial resolution of the lesions after two months (Figure 3) and total healing by the sixth month of treatment (Figure 4).

**Figure 5:**
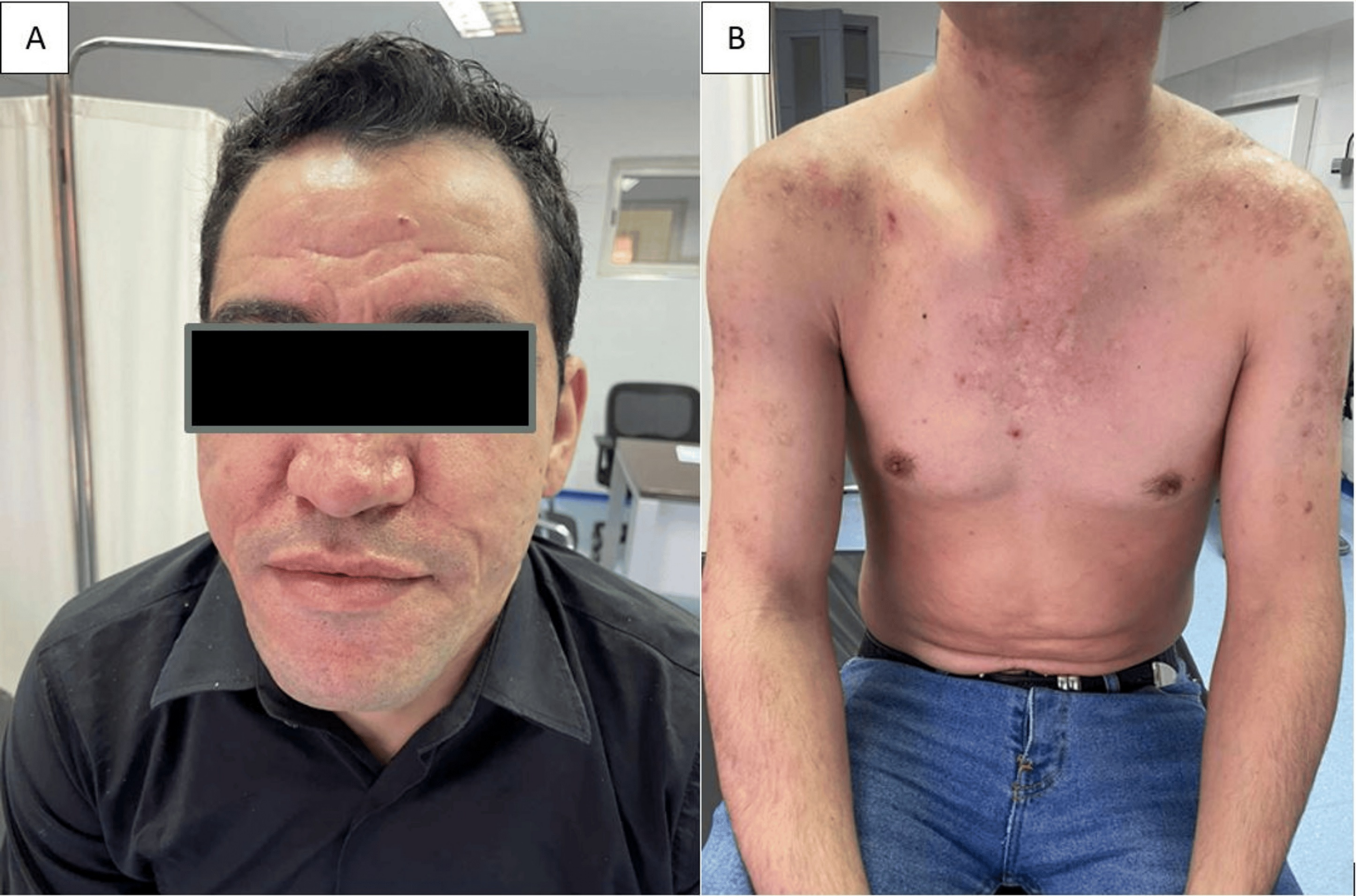
No recurrence after three years of treatment.

Given the clinical improvement observed, treatment was discontinued after six months, with no side effects or relapses after three years of biannual follow-up (Figure 5).

## Discussion

Acne fulminans is a severe form of acne, resulting from an excessive inflammatory response of the hair follicles and sebaceous glands; it is induced by hormonal and immune factors and is characterized by deep, painful inflammatory skin lesions, accompanied by systemic symptoms such as fever, weight loss, and arthralgias [4].

Joint involvement in acne fulminans is variable and may be associated with a number of syndromes, including SAPHO (synovitis, acne, pustulosis, hyperostosis, and osteitis) syndrome, in which severe acne and arthralgias are often associated with palmoplantar pustulosis, and PAPA syndrome, which combines acne, pyogenic arthritis, and gangrenous pyoderma [5].

First-line treatment of acne fulminans is based on corticosteroids (0.5-1mg/kg/day), which is then combined with low-dose isotretinoin (0.1mg/kg/day) [5]. However, in case of severe acne, isotretinoin treatment can paradoxically cause and worsen acne fulminans, and this phenomenon is explained by an excessive inflammatory and immune response [5,6].

In such cases, dapsone appears to be a good therapeutic alternative due to its bacteriostatic antibacterial activity by blocking folate synthesis as well as its immunomodulatory activity, which can be qualitative by altering polymorphonuclear neutrophils (PMNs) function [7] or quantitative by altering chemotaxis [8].

The dosage of dapsone varies according to age and individual susceptibility, ranging from 25mg to 100mg per day in adults, with a maximum dose of 2mg/kg/day in children as a single dose [3]. However, the topical form of dapsone (5% and 7.5% gel) is not recommended for the treatment of severe acne but rather for mild-to-moderate acne vulgaris [9]. However, treatment with systemic dapsone is not without risk and can cause a number of side effects (Table 2) [3,10].

**Table 2:** Classification of the side effects of dapsone. DRESS, drug reaction with eosinophilia and systemic symptoms

Mechanism	Adverse effects	Managed implications
Pharmacodynamics	Hemolysis, methemoglobinemia, macrocytosis, vitamin B9 and B12 deficiency	Pharmacodynamic reactions are dose-dependent and should be managed with dose reduction or discontinuation if severe
Idiosyncratic	Drug-induced skin reactions, particularly DRESS syndrome, digestive disorders, axonal polyneuritis, hepatitis, acute agranulocytosis.	Require immediate drug withdrawal
Exceptional	Nephrotic syndrome, renal papillary necrosis, autoimmune hypothyroidism, bone marrow aplasia, photodermatitis, eosinophilic pneumonia, psychiatric disorders	These effects appear to be rare but serious. Some may require discontinuation of treatment.

It is therefore essential to carry out a pre-therapeutic evaluation, including a complete blood count, renal and liver function tests, and a G6PD assay, in compliance with dapsone contraindications (Table 3) [3,10].

**Table 3:** Classification of contraindications for dapsone.

Contraindications for dapsone	
Absolute	Hypersensitivity to dapsone or sulfapyridine; severe methemoglobinemia; methemoglobin reductase deficiency; coronary, cardiac, or respiratory insufficiency limiting tolerance to anemia and methemoglobinemia; ischemic stroke; severe anemia; porphyria; breast-feeding
Relative	Hepatic or renal insufficiency (reduce the initial dose of dapsone), glutathione reductase deficiency, G6PD deficiency, primary or secondary hemosiderosis (risk of aggravation by chronic hemolysis and intake of iron oxalate), severe psychiatric condition

Unlike breast-feeding, pregnancy is not a contraindication to treatment, and the experience with dapsone in tens of thousands of pregnant women with leprosy is reassuring [11].

Rigorous clinical and biological monitoring is essential to ensure patient safety and consists of clinical surveillance for anemic syndrome, skin rash, and cyanosis suggestive of methemoglobinemia, and performing a neurological examination in the first month and quarterly thereafter [3].

Biologically, a reticulocyte count is required weekly for one month, then monthly for three months, and then quarterly, a methemoglobinemia test is required on day 8, and liver and kidney function tests are required after one month and then quarterly [3,10].

Our observation is consistent with previous studies, in which dapsone showed a marked improvement in the clinical condition of patients with severe acne, with a significant reduction in inflammatory lesions.

However, the available studies on its use in severe acne are relatively limited, small in sample size, and predominantly observational, and do not provide a therapeutic consensus [12-20]. Therefore, larger analytical studies are needed to define a specific consensus on the optimal doses and duration of treatment required and to confirm its long-term efficacy and safety.

## Conclusions

Acne is a complex condition, generally considered benign, but its psychological and social impact can be dramatic. While diagnosis is often straightforward, management can be difficult in some cases. Our observation highlights the efficacy and safety of dapsone in the treatment of acne fulminans, particularly in forms resistant to conventional therapies. However, further studies and longer-term follow-up are needed to confirm its performance in this specific indication.
